# Tireotoxicose Induzida pela Amiodarona - Revisão de Literatura e Atualização Clínica

**DOI:** 10.36660/abc.20190757

**Published:** 2021-11-01

**Authors:** Luciana Vergara Ferraz de Souza, Maria Thereza Campagnolo, Luiz Claudio Behrmann Martins, Maurício Ibrahim Scanavacca

**Affiliations:** 1 Faculdade de Medicina de Jundiaí Jundiaí SP Brasil Faculdade de Medicina de Jundiaí, Jundiaí, SP – Brasil; 2 Centro Universitário Lusiada Faculdade de Ciências Médicas de Santos Santos SP Brasil Centro Universitário Lusiada Faculdade de Ciências Médicas de Santos, Santos, SP – Brasil; 3 Universidade de São Paulo Instituto Instituto do Coração - Arrritmia e Marcapasso São Paulo SP Brasil Universidade de São Paulo Instituto do Coração - Arrritmia e Marcapasso, São Paulo, SP – Brasil

**Keywords:** Amiodarona/uso terapêutico, Arritmias Cardíacas, Iodo, Hipertireoidismo, Tireotoxicose, Hipotireoidismo, Tireotoxicose, Tireoidite

## Abstract

A amiodarona é amplamente utilizada no tratamento de arritmias atriais e ventriculares, porém devido sua alta concentração de iodo, o uso crônico da droga pode induzir distúrbios tireoidianos. A tireotoxicose induzida pela amiodarona (TIA) pode descompensar e exacerbar anormalidades cardíacas subjacentes, provocando aumento da morbidade e mortalidade, principalmente em pacientes com fração de ejeção do ventrículo esquerdo <30%.

Os casos de TIA são classificados em dois subtipos que direcionam a conduta terapêutica. Os riscos e benefícios de manter a amiodarona devem ser avaliados de maneira individualizada, e a decisão de continuar ou suspender a droga deve ser tomada conjuntamente por cardiologistas e endocrinologistas.

O tratamento de TIA tipo 1 é semelhante ao do hipertireoidismo espontâneo, sendo indicado o uso de drogas antitireoidianas (metimazol e propiltiouracil) em doses elevadas. A TIA tipo 1 mostra-se mais complicada, pois apresenta proporcionalmente maiores números de recorrências ou até mesmo a não remissão do quadro, sendo recomendado o tratamento definitivo (tireoidectomia total ou radioiodo).

TIA tipo 2 é geralmente autolimitada, mas devido a elevada mortalidade associada a tireotoxicose em pacientes cardiopatas, o tratamento deve ser instituído para que o eutireoidismo seja atingido mais rapidamente. Em casos bem definidos de TIA tipo 2, o tratamento com corticosteroides é mais efetivo do que o tratamento com drogas antitireoidianas.

Em casos graves, independentemente do subtipo, a restauração imediata do eutiroidismo por meio da tireoidectomia total deve ser considerada antes que o paciente evolua com piora clínica excessiva, pois a demora na indicação da cirurgia está associada ao aumento da mortalidade.

## Introdução

Amiodarona é uma droga antiarrítmica classe III frequentemente utilizada no tratamento de arritmias atriais e ventriculares,^1^ principalmente quando são refratárias a outras drogas antiarrítmicas.^2^ É também utilizada na profilaxia da morte súbita de causa cardíaca em pacientes de alto risco, sobretudo em pacientes sem acesso ao cardioversor desfibrilador implantável, apresentando redução da mortalidade quando comparada a placebo e outros antiarrítmicos.^3^

Devido à sua alta concentração de iodo, a amiodarona pode induzir disfunção tireoidiana (hipertireoidismo ou hipotireoidismo) em até 36% dos pacientes que fazem uso crônico desta medicação.^4,5^ A incidência de hipertireoidismo varia de 2% a 18%,^4-12^ e a de hipotireoidismo de 5%-22% (Tabela 1).^4-10,12^ A influência do iodo no desenvolvimento desses distúrbios tireoidianos é tamanha que, de acordo com seu consumo alimentar regional, percebe-se uma mudança na forma como a amiodarona altera o comportamento da tireoide. Proporcionalmente, nas áreas onde o consumo de iodo é elevado predominam os casos de hipotireoidismo induzido pela amiodarona, enquanto em locais de baixa ingestão a incidência de tireotoxicose induzida pela amiodarona (TIA) é maior.^4,6,8^

**Tabela 1:** Estudos demonstrando incidência dos distúrbios tireoidianos induzidos pelo uso da amiodarona

Primeiro autor, Ano	País	Número de pacientes	Hipotireoidismo induzido pela amiodarona	Tireotoxicose induzida pela amiodarona	Tipo de estudo
Martino E,8 1984	Itália E.U.A.	Itália: 188 E.U.A.: 41	Itália 10 (5%) E.U.A.: 9 (22%)	Itália: 18 (9.6%) E.U.A.: 1 (2%)	Não descrito
Trip MD,^4^ 1991	Países Baixos	58	10 (17,2%)	11 (18,9%)	Prospectivo
Yiu KH,^5^ 2009	Hong Kong	354	73 (20.6%)	57 (16.1%) TIA 1: 5/57 TIA 2: 13/57 Mista/incerta: 35/57	Prospectivo 2000-2005
Stan MN,^25^ 2013	E.U.A.	169	Não estudado	23 (13,6%) TIA 1: 7/23 TIA 2: 13/23 TIA mista/incerta: 3/23	Retrospectivo 1987-2009 Adultos com doença cardíaca congênita
Huang C-J,^12^ 2014	Taiwan	527	69 (13.1%)	21 (4%)	Retrospectivo 2008-2009
Uchida T,^11^ 2014	Japão	225	Não estudado	13 (5.8%) TIA 2	Retrospectivo 2008-2012
Lee KF,^9^ 2010	Hong Kong	390	87 (22%)	24 (6%)	Retrospectivo 2005-2007
Benjamens S,^6^ 2017	Países Baixos	303	33 (10,8%)	44 (15,5%)	Retrospectivo 1984-2007
Barrett B,^10^ 2019	E.U.A.	190	26 (13.7%)	4 (2.1%) 25% resolução espontânea	Retrospectivo 2007-2018 Crianças e adultos jovens

O hipotireoidismo induzido pela amiodarona é de menor gravidade que o hipertireoidismo e tem tratamento mais simples. Nos casos de hipotireoidismo não é necessária a retirada da amiodarona, e o tratamento pode ser feito apenas com a introdução da levotiroxina. Em alguns casos subclínicos o ajuste (redução) da dosagem da amiodarona pode ser suficiente para que ocorra a normalização da função tireoidiana. Portanto, nos pacientes subclínicos não é necessária a reposição hormonal, apenas a análise regular da função tireoidiana para avaliar a progressão para hipotireoidismo.^13,14^

Clinicamente, quadros de TIA oferecem riscos maiores de complicações, além do diagnóstico e tratamento serem muito mais complexos. A exposição prolongada a altos níveis de hormônios tireoidianos pode levar ao aparecimento de arritmias e a uma rápida deterioração da função cardíaca.^5,15^ Um estudo observacional analisando 354 pacientes em uso crônico de amiodarona demonstrou um aumento significativo de eventos cardiovasculares maiores no grupo que desenvolveu TIA, comparado ao grupo que permaneceu eutireoideo (31,6% vs. 10,7%, p<0.01), especialmente devido à alta incidência de arritmias ventriculares com necessidade de hospitalização (7% vs. 1,3%, p = 0.03).^5^ Outro estudo relatou uma taxa de mortalidade de 10% antes do controle da tireotoxicose, associada a fração de ejeção do ventrículo esquerdo (FEVE) <30%.^15^

Com base em estudos e diretrizes recentes, foram revisados e sintetizados de forma prática os principais aspectos diagnósticos e terapêuticos da TIA. Também, destaca-se a importância das decisões terapêuticas a serem tomadas em conjunto entre cardiologistas e endocrinologistas.

## Métodos

Foi realizada uma revisão da literatura através de busca na MEDLINE utilizando as combinações dos termos MeSH: “Amiodarone”, “Thyrotoxicosis” e “Thyroid”. Também foram feitas buscas manuais e eletrônicas de referências citadas nos estudos avaliados. Foram incluídos estudos clínicos que abordam alterações tireoidianas secundárias ao uso da amiodarona, com foco na incidência e no tratamento clínico e cirúrgico. Foram excluídos os trabalhos que abordam transtornos causados pela amiodarona em outros órgãos e relatos de caso com número inferior a dez pacientes. Nos dados compilados também foram analisados os consensos mais atuais da Sociedade Brasileira de Endocrinologia e Metabologia (SBEM), Associação Americana de Tireoide (ATA) e Associação Europeia de Tireoide (ETA).

### Amiodarona: mecanismo de ação sobre a tireoide

A amiodarona pode agir influenciando a glândula tireoide de diversas maneiras. Estruturalmente, a amiodarona é uma medicação diiodinada, sendo 37% do seu peso molecular referente ao iodo, portanto a cada 200mg de amiodarona (dose diária de manutenção) cerca de 7.5mg de iodo são liberadas. A dose diária de iodo recomendada pela Organização Mundial de Saúde é de 0,15mg (adultos),^16^ e com o uso da amiodarona, cerca de 7,5mg de iodo é liberado na forma livre no organismo diariamente, excedendo a dose recomendada em 50 vezes.^17^

A medicação também possui extrema similaridade com os hormônios triiodotironina (T3) e tiroxina (T4),^18^ e sua longa meia-vida garante a permanência da substância no organismo por até 100 dias, o que potencializa sua toxicidade e permite que os efeitos colaterais ocorram durante o uso e mesmo após a suspensão do medicamento.^19-21^

Apesar do reconhecimento de que a medicação influencia a tireoide propriamente dita e a metabolização de seus hormônios no organismo, ainda são escassas informações a respeito do seu mecanismo de ação. A inibição da enzima 5’-deiodinase é uma das teorias sobre a forma pela qual a amiodarona age sobre o metabolismo dos hormônios tireoidianos. Essa interação resulta em aumento sérico do T3 reverso e T4, substratos da enzima em questão, concomitantemente com a diminuição do T3, produto da conversão realizada pela molécula inibida. A sobrecarga de iodo e a citotoxicidade induzidas pelo medicamento também corroboram a explicação do surgimento de distúrbios tireoidianos como efeitos colaterais do uso crônico da medicação.^18,22^

### Tireotoxicose induzida pela amiodarona (TIA)

A TIA está associada a altas taxas de eventos cardiovasculares maiores e ao aumento da mortalidade, principalmente da morte cardiovascular. O surgimento ou recorrência de arritmias ventriculares e a disfunção ventricular esquerda severa (FEVE <30%) são os principais fatores relacionados a este aumento.^5,14,15^ Portanto, a restauração do eutireoidismo deve ser estabelecida o quanto antes, e em casos emergenciais a tireoidectomia pode ser indicada para uma resolução rápida da tireotoxicose.^14,19,23,24^

**Figura 1:**
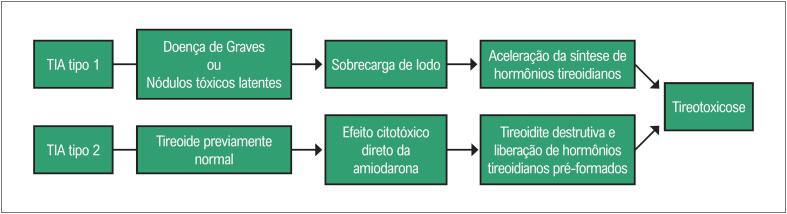
Fisiopatologia das principais formas de TIA. TIA: tireotoxicose induzida pela amiodarona.

Os casos de TIA são divididos em dois subtipos devido a diferenças na fisiopatologia e a necessidade de tratamento direcionado. A TIA tipo 1 (TIA 1) tem lugar por meio da produção autônoma de hormônios tireoidianas devido à sobrecarga de iodo, particularmente concomitante às alterações prévias da tireoide (nódulos tireoidianos ou doença de Graves latente). A TIA tipo 2 (TIA 2) é a forma mais frequente e ocorre em pacientes com a tireoide previamente saudável, correspondendo a uma tireoidite destrutiva devido a citotoxicidade direta da amiodarona nas células foliculares, com consequente liberação das reservas hormonais pré-formadas e indução da tireotoxicose.^11,14,19,24,25^ A figura 1 ilustra as diferenças na fisiopatologia dos dois subtipos. Ocasionalmente essa distinção é complicada essa distinção é complicada e existe sobreposição dos dois subtipos, e esses casos são denominados como formas mistas ou indefinidas.^13,14,19^

### Diagnóstico

Os meios para a identificação de pacientes sob maior risco de desenvolverem disfunção tireoidiana secundária ao uso da amiodarona ainda não foram definidos.^14,26^ A Associação Americana de Tireoide recomenda avaliação da função tireoidiana por meio da dosagem sérica de tireotropina (TSH) e T4 livre. A função tireoidiana deve ser avaliada antes de iniciar a amiodarona, dentro dos primeiros três meses após o início da droga, e posteriormente a cada três a seis meses.^24^

Inicialmente, pacientes tratados com amiodarona apresentam alterações da função tireoidiana, porém a maioria retorna à normalidade sem a necessidade de tratamento ou descontinuação da droga. Nos três primeiros meses de tratamento com amiodarona, ocorre aumento dos níveis séricos de TSH, T4 e T3 reverso, e concomitante diminuição de T3. Posteriormente, ocorre normalização dos níveis de TSH, T4 e T3, podendo o T4 manter-se no limite superior de normalidade ou discretamente elevado, e o T3 reverso permanecer elevado.^14^

A dosagem de TSH é o método mais sensível e específico para o diagnóstico de hipertireoidismo, pois pequenas alterações nos níveis de T4 livre provocam mudanças expressivas nas concentrações de TSH. No hipertireoidismo subclínico, os níveis de TSH encontram-se baixos ou até indetectáveis, e os valores de T4 livre e T3 apresentam-se normais.^24^ Na tireotoxicose, o TSH encontra-se muito baixo ou indetectável, e os níveis de T4 livre e/ou T3 elevados.^19,24^

Os pacientes com TIA podem ser assintomáticos ou apresentar um quadro clínico típico de hipertireoidismo, como sintomas de palpitações, tremores, sudorese, intolerância ao calor, nervosismo e perda de peso. O bloqueio beta adrenérgico da amiodarona no coração pode justificar a ausência de palpitações, o que torna a apresentação clínica de TIA ainda mais insidiosa.^17^ O diagnóstico de tireotoxicose é confirmado pelos níveis séricos de TSH suprimidos e níveis elevados dos hormônios tireoidianos T3 e T4 livres.^14,19^

A diferenciação entre os dois subtipos de TIA pode ser difícil, no entanto, alguns parâmetros laboratoriais associados à ultrassonografia de tireoide com Dopplerfluxometria podem ser usados para a distinção apropriada.^13,14,19,24^ As características dos subtipos de TIA estão sintetizadas na Tabela 2.

**Tabela 2:** Principais características dos subtipos de TIA^14^

Características	TIA tipo 1	TIA tipo 2
Alterações tireoidianas prévias	Sim	Usualmente não
Doppler fluxometria	Vascularidade aumentada	Ausência de hipervascularidade
Captação de iodo radioativo	Baixa, normal ou elevada	Suprimida
Anticorpos antitireoidianos	Presente se relacionado a doença de Graves	Usualmente ausentes
Início após amiodarona	Curto (média de 3 meses)	Longo (média de 30 meses)
Remissão espontânea	Não	Possível
Evolução para hipotireoidismo	Não	Possível
Tratamento de primeira linha	Drogas antitireoidianas	Glicocortocoides orais
Tratamento definitivo subsequente	Geralmente sim	Não

Modificado de Bartalena L et al.,^14^ TIA: tireotoxicose induzida pela amiodarona.

Acreditava-se que o nível sérico de interleucina-6 se apresentava altamente elevado nos casos de TIA 2 e assim seria útil na diferenciação dos subtipos de TIA, porém há uma sobreposição entre os subtipos e, portanto, não pode ser utilizado para a distinção.^24,27^ A captação de radioiodo (^131^I ou ^123^I) é útil nessa diferenciação em áreas de baixa ingestão de iodo, pois nessas regiões os pacientes com TIA 2 apresentam captação suprimida de radioiodo. Na TIA 1 a captação pode ser baixa, normal ou até elevada. Todavia, em áreas com ingestão suficiente de iodo, caso da maior parte das regiões metropolitanas do Brasil, a captação de radioiodo encontra-se sempre suprimida tornando a investigação inútil.^14,24,28^

A detecção de anticorpos anti-tireoperoxidase (anti-TPO)^14,24^ e a presença de bócios difusos ou nodulares na ultrassonografia de tireoide apontam para TIA 1,^21,23^ contudo devido a sua elevada prevalência na população esses achados também não excluem a TIA 2.^13,14,24^ Diversos estudos recentes indicam que a ausência de hiperfluxo na dopplerfluxometria é sugestiva de TIA 2.^19,24,27,28^ Estes achados não devem ser utilizados de forma isolada devido a possibilidade de formas mistas.^14^

### Manter ou suspender a amiodarona?

A necessidade da retirada da amiodarona ainda é controversa. Em muitos casos, ela é a única medicação capaz de controlar a arritmia cardíaca, e devido a sua meia-vida prolongada, a retirada não traria benefícios imediatos.^14^ Além disso, é importante salientar que alguns pacientes possuem recorrência dos distúrbios tireoidianos, mesmo meses após a suspensão de amiodarona. Ademais, a droga apresenta propriedades antagonistas de T3 e inibe a conversão de T4 para T3 no coração, portanto a sua suspensão poderia agravar as manifestações clínicas.^20,21,24^

A TIA 2 é geralmente autolimitada, e a amiodarona pode ser mantida nesses pacientes.^14,29-32^ Estudos observacionais com pacientes TIA 2 revelaram que os pacientes retornam ao eutireoidismo mesmo mantendo a amiodarona.^29,30,31^ No entanto, estudos mostram uma variação de 8% a 73% de recorrência da tireotoxicose em pacientes que mantiveram o uso da medicação.^29,31,33,34^ Um estudo com 10 anos de seguimento envolvendo 50 pacientes que mantiveram amiodarona apresentou apenas três casos de recorrência da tireotoxicose, sendo muito mais brandos do que no primeiro episódio.^32^

A decisão de retirar a amiodarona deve ser individualizada e tomada em conjunto pelo cardiologista e pelo endocrinologista, levando em consideração os riscos e benefícios da retirada da droga.^14,19,24^ É amplamente aceito que se continue a medicação em pacientes críticos com arritmias que ameaçam a vida e que apresentem boa resposta cardíaca a droga.^14,24,32^ Se as condições cardíacas estiverem estáveis e houver uma alternativa segura, a amiodarona pode ser descontinuada.^13,14^

### Tratamento

Em pacientes clinicamente estáveis com evidência que diferencia o subtipo, o tratamento deve ser estabelecido de acordo com o subtipo no qual o paciente se encaixa.^14,19,24^ Nos quadros de tireotoxicose moderada com comprometimento da função cardíaca, a Associação Americana de Tireoide recomenda iniciar terapia combinada com drogas antitireoidianas e corticosteroides.^24^

**Figura 2:**
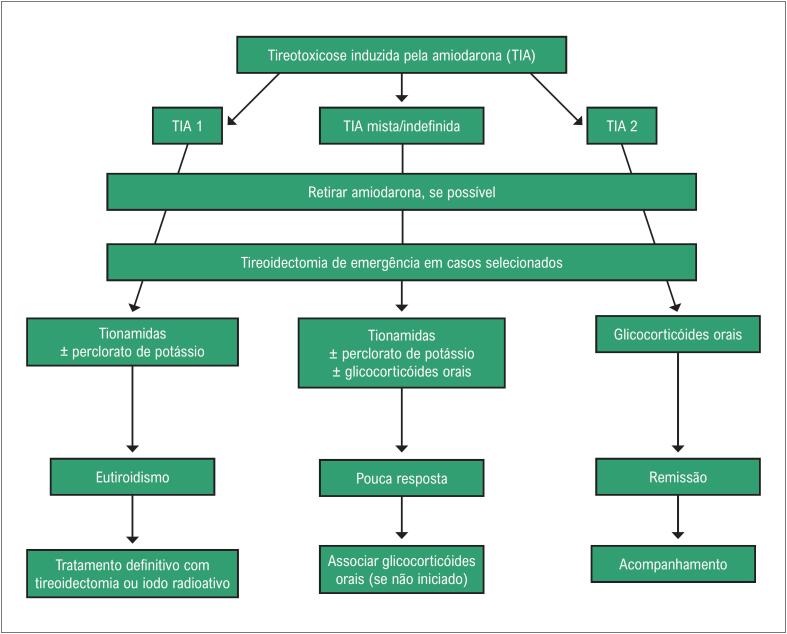
Algoritmo para manejo de tireotoxicose induzida pela amiodarona (TIA). Modificado de Bartalena L, et al.^14^

Se o paciente apresentar deterioração rápida da função cardíaca, tireoidectomia de emergência deve ser feita independentemente do subtipo TIA.^14,24^ A Figura 2 mostra o algoritmo para manejo de TIA conforme proposto pela Associação Europeia de Tireoide.^14^ Como nos casos de TIA a tireoide encontra-se repleta de iodo, o tratamento com iodo radioativo é inviável por pelo menos seis a nove meses a partir da suspensão da droga.^13,19,20^

### Tratamento: TIA 1

O tratamento de TIA 1 é feito com drogas antitireoidianas (DAT), mas estas são menos efetivas devido à alta concentração de iodo, e se faz necessário o uso de doses maiores (40-60mg/dia de metimazol ou doses equivalentes de propiltiouracil).^14,24^ Se o paciente permanecer estável, a DAT deve ser mantida até a restauração do eutireoidismo,^14,19,20,35^ geralmente entre três e seis meses.^24^

O perclorato de potássio pode ser associado nas primeiras semanas para diminuir a captação de iodo pela tireoide e tornar a tireoide mais sensível as DAT.^1,14,19,35,36^ Devido a sua toxicidade, este não deve exceder 1g/dia e não deve ser mantido por mais de 4-6 semanas.^1,14^

A tireotoxicose pode recorrer ou até não entrar em remissão, e nesses casos o tratamento definitivo é recomendado.^14,19,36^ Se a amiodarona for descontinuada, o tratamento definitivo com radioiodo pode ser feito após seis a nove meses. A tireoidectomia deve ser considerada se não for possível a retirada da amiodarona.^14,19^ De uma forma geral, o tratamento definitivo de TIA 1 é semelhante ao do hipertireoidismo espontâneo.^14^

### Tratamento: TIA 2

A TIA 2 é geralmente autolimitada, entretanto devido ao aumento da mortalidade associada a tireotoxicose em pacientes cardiopatas, o tratamento deve ser instituído para que o eutireoidismo seja atingido mais rapidamente.^14,19,36^ A decisão de tratar casos leves ou subclínicos deve ser feita levando em consideração as alterações cardíacas do paciente.^14^

Tem sido sugerido que, em casos bem definidos de TIA 2, o tratamento com corticosteroides é mais efetivo do que o tratamento com DAT.^29,37^ As doses utilizadas são de 30-40mg/dia de prednisona ou dose equivalente de outro glicocorticoide por dois a três meses, com posterior retirada gradual baseada na resposta clínico.^14,24^ Em casos graves, assim como nos casos de TIA 1 e mista/indefinida, a tireoidectomia radical deve ser considerada.^8,14,38^

### Tratamento: TIA mistas ou indefinidas

Formas mistas ou indefinidas ainda não estão completamente caracterizadas. Acredita-se que esses casos envolvam mecanismos patogênicos mistos dos dois subtipos, tanto de aumento da produção hormonal quanto por tireoidite destrutiva.^14,36^

O tratamento das formas mistas ou indefinidas deve ser feito com DAT, podendo ser associados corticosteroides orais no início do tratamento, ou após 4-6 semanas se a resposta for pequena.^14,19,35^ Em casos mais severos, a terapia combinada com DAT e corticosteroides deve ser iniciada prontamente.^24^

### Tratamento: Tireoidectomia

À tireoidectomia total corresponde à melhor opção em pacientes cujo tratamento clínico é falho ou naqueles em que há demora de resposta terapêutica associada a função ventricular deprimida, sendo a melhor alternativa para restauração imediata do eutireoidismo.^14,39,40^ Apesar dos riscos associados à tireoidectomia, esta deve ser considerada antes que o paciente evolua com piora clínica severa, pois a demora na indicação da cirurgia está associada ao aumento da mortalidade.^24,39-42^ Diversos estudos avaliando pacientes com TIA submetidos à tireoidectomia relataram baixa morbidade associada ao procedimento, apresentando mortalidade de 0% a 1.9%.^40-44^

Um estudo observacional recente, 207 pacientes com TIA (57 tireoidectomizados, 156 tratamento clínico), evidenciou menor mortalidade nos pacientes submetidos a tireoidectomia comparada aos tratados apenas clinicamente, particularmente em pacientes com FEVE <40%. Neste mesmo estudo foi demonstrada uma melhora significativa da FEVE após a restauração do eutireoidismo, sendo mais evidente nos pacientes com FEVE <40%.^42^ Outros três estudos também relataram melhora significativa da função cardíaca após tireoidectomia, sendo três pacientes retirados da lista de transplante cardíaco após restauração do eutireoidismo.^40,41,43^

Se a tireoidectomia total for considerada, a avaliação individualizada dos riscos e benefícios deve ser feita, e a decisão deve ser multidisciplinar, envolvendo cardiologistas, endocrinologistas, cirurgiões e anestesistas. É imprescindível que um cirurgião com alto volume operatório e experiência com tireoidectomias seja o responsável pelo procedimento.^14^

Tireoidectomia total deve ser considerada quando há:^14,24,39,43^:

– Resposta insuficiente ao tratamento medicamentoso com DAT e corticosteroides;
– Deterioração rápida da função cardíaca;
– Doença cardíaca avançada, displasia arritmogênica ventricular direita, e arritmias malignas;
– Tratamento definitivo alternativo ao radioiodo;

## Conclusão

Dadas as consequências acarretadas pela TIA, salienta-se a importância de diagnosticar e tratar os subtipos de TIA conjuntamente. Enfatiza-se a importância de as decisões terapêuticas serem tomadas de forma conjunta por cardiologistas e endocrinologistas, e que nos casos mais severos a tireoidectomia deve ser considerada antes que ocorra piora clínica exagerada.

Estudos clínicos envolvendo pacientes com TIA ainda são limitados e insuficientes, principalmente ensaios clínicos randomizados multicêntricos. Visto que a amiodarona é uma droga bastante utilizada, e devido as consequências da TIA, destaca-se a necessidade de novos ensaios clínicos para aprimorar o manejo destes pacientes.
