# Supplementary material for: Tireotoxicose Induzida pela Amiodarona - Revisão de Literatura e Atualização Clínica
Source: Arq Bras Cardiol. 2021 Nov 1;117(5):1038–44. [Article in Portuguese] doi: 10.36660/abc.20190757 (PMC8682089; doi:10.36660/abc.20190757)
Supplement: Translation

# Translation: Amiodarone-Induced Thyrotoxicosis - Literature Review & Clinical Update

**Authors:** Luciana Vergara Ferraz de Souza, Maria Thereza Campagnolo, Luiz Claudio Behrmann Martins, Maurício Ibrahim Scanavacca

**Affiliations:** 1 Jundiaí SP Brazil Faculdade de Medicina de Jundiaí, Jundiaí, SP – Brazil; 2 Santos SP Brazil Centro Universitário Lusiada Faculdade de Ciências Médicas de Santos, Santos, SP – Brazil; 3 São Paulo SP Brazil Universidade de São Paulo Instituto do Coração - Arrritmia e Marcapasso, São Paulo, SP – Brazil

**Keywords:** Amiodarone/therapeutic use, Arrhythmias,Cardiaca, Iosine, Hyperthyroidism, Thyrotoxicosis, Hypothyroidis, Thyrotoxicosis, Thyroiditis

## Abstract

Amiodarone is widely used in treating atrial and ventricular arrhythmias; however, due to its high iodine concentration, the chronic use of the drug can induce thyroid disorders. Amiodarone-induced thyrotoxicosis (AIT) can decompensate and exacerbate underlying cardiac abnormalities, leading to increased morbidity and mortality, especially in patients with left ventricular ejection fraction <30%.

AIT cases are classified into two subtypes that guide therapeutic management. The risks and benefits of maintaining the amiodarone must be evaluated individually, and the therapeutic decision should be taken jointly by cardiologists and endocrinologists.

Type 1 AIT treatment is similar to that of spontaneous hyperthyroidism, using antithyroid drugs (methimazole and propylthiouracil) at high doses. Type 1 AIT is more complicated since it has proportionally higher recurrences or even non-remission, and definitive treatment is recommended (total thyroidectomy or radioiodine).

Type 2 AIT is generally self-limited, yet due to the high mortality associated with thyrotoxicosis in cardiac patients, the treatment should be implemented for faster achievement of euthyroidism. Furthermore, in well-defined cases of type 2 AIT, the treatment with corticosteroids is more effective than treatment with antithyroid drugs.

In severe cases, regardless of subtype, immediate restoration of euthyroidism through total thyroidectomy should be considered before the patient progresses to excessive clinical deterioration, as delayed surgery indication is associated with increased mortality.

## Introduction

Amiodarone is a class III antiarrhythmic drug frequently used to treat atrial and ventricular arrhythmias,^1^ especially when refractory to other antiarrhythmic drug.^2^ It is also used for prophylaxis of sudden cardiac death in high-risk patients, mainly in patients without access to the implantable cardioverter-defibrillator, showing a reduction in mortality when compared to placebo and other antiarrhythmic drugs.^3^

Due to its high iodine concentration, amiodarone can induce thyroid dysfunction (hyperthyroidism or hypothyroidism) in up to 36% of patients who take this medication chronically.^4,5^ The incidence of hyperthyroidism ranges from 2% to 18%,^4-12^ and hypothyroidism from 5% to 22% (Table 1).^4-10,12^ The influence of iodine on the development of these thyroid disorders is so evident that, according to its regional dietary intake, there is a change in the way the amiodarone alters the behavior of the thyroid. Proportionally, in areas with high iodine intake, there is a predominance of amiodarone-induced hypothyroidism cases, while in places with low intake, there is a higher incidence of amiodarone-induced thyrotoxicosis (AIT).^4,6,8^

**Table 1:** Studies showing the incidence of thyroid disorders induced by amiodarone use

First Author, Year	Country	Number of patients	Amiodarone-induced hypothyroidism	Amiodarone-induced thyrotoxicosis	Study design
Martino E,8 1984	Italy U.S.A.	Italy: 188 U.S.A.: 41	Italy: 10 (5%) U.S.A.: 9 (22%)	Italy: 18 (9.6%) U.S.A.: 1 (2%)	Not described
Trip MD,^4^ 1991	The Netherlands	58	10 (17,2%)	11 (18,9%)	Prospective
Yiu KH,^5^ 2009	Hong Kong	354	73 (20.6%)	57 (16.1%) AIT 1: 5/57 AIT 2: 13/57 Mixed/uncertain: 35/57	Retrospective 2000-2005
Stan MN,^25^ 2013	U.S.A.	169	Not studied	23 (13,6%) AIT 1: 7/23 AIT 2: 13/23 Mixed/Undefined: 3/23	Retrospective 1987-2009 Adults with congenital heart disease
Huang C-J,^12^ 2014	Taiwan	527	69 (13.1%)	21 (4%)	Retrospective 2008-2009
Uchida T,^11^ 2014	Japan	225	Not studied	13 (5.8%) AIT 2	Retrospective 2008-2012
Lee KF,^9^ 2010	Hong Kong	390	87 (22%)	24 (6%)	Retrospective 2005-2007
Benjamens S,^6^ 2017	The Netherlands	303	33 (10,8%)	AIT: 44 (15,5%)	Retrospective 1984-2007
Barrett B,^10^ 2019	U.S.A.	Total: 190	26 (13.7%)	4 (2.1%) 25% spontaneous resolution	Retrospective 2007-2018 Pediatric and young adults

Amiodarone-induced hypothyroidism is less severe than hyperthyroidism and has a simpler treatment. In hypothyroidism cases, amiodarone withdrawal is unnecessary, and treatment can be done just by introducing levothyroxine. In some subclinical cases, the dose adjustment (reduction) may be enough for the thyroid function to return to normal. Therefore, there is no need for hormone replacement in subclinical patients, only regular thyroid function assessment to evaluate progression to hypothyroidism.^13,14^

Clinically, AIT cases pose greater complications risks; moreover, the diagnosis and treatment are far more complex. Prolonged exposure to high levels of thyroid hormones may lead to the onset of arrhythmias and rapid deterioration of cardiac function.^5,15^ An observational study analyzed 354 patients in chronic use of amiodarone demonstrated a significant increase in major cardiovascular events in the group that developed AIT, comparing to the group that remained euthyroid (31,6% vs. 10.7%, p<0.01), mainly due to the high incidence of ventricular arrhythmias leading to hospital admission (7% vs. 1.3%, p=0.03).^5^ Another study reported a 10% mortality rate before thyrotoxicosis control, associated with left ventricular ejection fraction (LVEF) <30%.^15^

AIT’s main diagnosis and treatment issues were revised and summarized practically based on recent studies and guidelines. Likewise, we emphasize the importance of therapeutic decisions to be taken jointly by cardiologists and endocrinologists.

## Methods

A literary review through MEDLINE search using the combinations of the MeSH terms: “Amiodarone”,”Thyrotoxicosis”, and “Thyroid” was conducted. Also, manual and electronic searches were performed based on references cited in the studies evaluated. Clinical studies that address thyroid changes secondary to amiodarone use, focusing on the incidence and the clinical and surgical treatment, were included. Were excluded studies that addressed other organs disorders caused by amiodarone and case reports with less than ten patients. In the compiled data were also analyzed the most current consensus of the Brazilian Society of Endocrinology and Metabology (SBEM), American Thyroid Association (ATA) and European Thyroid Association (ETA).

### Amiodarone: mechanism of action on the thyroid

In many ways, amiodarone can act by influencing the thyroid gland. Structurally, amiodarone is a diiodinated medication, with 37% of its molecular weight referring to iodine; thus, each 200mg of amiodarone (daily maintenance dose) contains about 75mg of iodine. The daily dose of iodine recommended by the World Health Organization is 0.15mg (adults),^16^ and with the use of amiodarone, about 7.5mg of free iodine are released in the body daily, exceeding the recommended dose by 50 times.^17^

The medication also has extreme similarity with the hormones triiodothyronine (T3) and thyroxine (T4),^18^ and its long half-life ensures that the substance stays in the body for up to 100 days, which enhances its toxicity and allows the side effects to occur during its use and even after the drug withdrawal.^19-21^

Despite the recognition that medication influences the thyroid itself and the metabolism of its hormones in the body, there is still little information about its mechanism of action. Inhibition of the enzyme 5’-deiodinase is one of the theories about how amiodarone acts on thyroid hormone metabolism. This interaction results in a serum increase of the reverse T3 and T4 substrates of the enzyme in question, concurrently with the decrease of T3, a product of the conversion performed by the inhibited molecule. Iodine overload and drug-induced cytotoxicity also corroborate the explanation of the onset of thyroid disorders as side effects of chronic medication use.^18,22^

### Amiodarone-induced Thyrotoxicosis (AIT)

AIT is associated with high rates of major cardiovascular events and increased mortality, principally cardiovascular death. The appearance and recurrence of ventricular arrhythmias and severe left ventricular dysfunction (LVEF <30%) are the main factors related to this increase.^5,14,15^ Therefore, restoration of euthyroidism should be established as soon as possible, and in emergency cases, the thyroidectomy can be indicated for a rapid resolution of the thyrotoxicosis.^14,19,23,24^

AIT cases are divided into two subtypes due to differences in pathophysiology and the need for directed treatment. Type 1 AIT (AIT 1) occurs through autonomous production of thyroid hormones due to iodine overload, particularly with concomitant previous thyroid abnormalities (thyroid nodules or latent Graves’ disease). Type 2 AIT (AIT 2) is the most frequent and occurs in patients with a previously healthy thyroid, corresponding to destructive thyroiditis due to direct amiodarone cytotoxicity in the follicular cells, with consequent release in the hormonal reserves preformed and the thyrotoxicosis induction.^11,14,19,24,25^ Figure 1 illustrates the differences in pathophysiology of the two subtypes. Occasionally, this distinction is complicated, and there is an overlap between the two subtypes; these cases are denominated as mixed or undefined forms.^13,14,19^

**Figure 1:** Pathophysiology of the main forms of AIT. AIT: amiodarone-induced thyrotoxicosis.

### Diagnosis

Means to identify patients at higher risk of developing thyroid dysfunction secondary to amiodarone use have not yet been defined.^14,26^ The American Thyroid Association recommends thyroid function evaluation through serum dosage of thyrotropin (TSH) and free T4 evaluation before starting amiodarone and every three to six months during the medication use. The thyroid function should be evaluated before starting amiodarone, within the first three months after its initiation, and then every three to six months.^24^

Initially, patients treated with amiodarone present thyroid function changes; however, the majority return to normal without the need for treatment or drug discontinuation. In the first three months of treatment with amiodarone, there is a rise in the serum levels of TSH, T4 and reverse T3, and a concomitantly decrease of T3. After that, the levels of TSH, T4 and T3 normalize, and T4 may remain at the upper limit of normality or slightly elevated, and the reverse T3 remains elevated.^14^

TSH measurement is the most sensitive and specific method for diagnosing hyperthyroidism, as small changes in free T4 levels cause significant changes in TSH concentrations. In subclinical hyperthyroidism, TSH levels are low or even undetectable, and free T4 and T3 values are norma.^24^ In thyrotoxicosis, TSH is very low or undetectable, and free T4 and/or T3 levels are elevated.^19,24^

The patients with AIT may be asymptomatic or have the typical clinical picture of hyperthyroidism, with symptoms such as palpitations, tremors, sweating, heat intolerance, nervousness and weight loss. Amiodarone beta-adrenergic blockade in the heart may justify the absence of palpitations, which makes AIT clinical presentation even more insidious.^17^ The diagnosis of thyrotoxicosis is confirmed by suppressed serum TSH levels and elevated levels of free T3 and free T4 thyroid hormones.^14,19^

The differentiation between the two AIT subtypes can be difficult, although some laboratory parameters associated with thyroid ultrasound with Dopplerfluxometry may be used for the proper distinction.^13,14,19,24^ The characteristics of AIT subtypes are summarized in Table 2.

**Table 2:** Main characteristics of AIT subtypes^14^

Characteristics	Type 1 AIT	Type 2 AIT
Previous thyroid changes	Yes	Usually no
Dopplerfluxometry	Increased vascularity	No hypervascularity
Radioactive iodine uptake	Low, normal or high	Supressed
Antithyroid Antibodies	Present if related to Graves' disease	Usually absent
Onset time after starting amiodarone	Short (median 3 months)	Long (median 30 months)
Spontaneous remission	No	Possible
Subsequent hypothyroidism	No	Possible
First-line treatment	Antithyroid Drugs	Oral glucocorticoids
Subsequent definitive treatment	Generally yes	No

Modified from Bartalena L et al.^14^ AIT: amiodarone-induced thyrotoxicosis.

It was believed that the serum interleukin-6 level was highly elevated in AIT 2 and, therefore, valuable for differentiating AIT subtypes; however, there is an overlap between AIT subtypes; thus, it can not be used for this distinction.^24,27^ Radioiodine uptake (^131^I or ^123^I) is helpful in this differentiation in areas of low iodine intake, as in these regions, patients with AIT 2 present suppressed radioiodine uptake. In AIT 1, the uptake may be low, normal or even high. However, in areas with sufficient iodine intake, as in most metropolitan regions of Brazil, radioiodine uptake is always suppressed, making the investigation useless.^14,24,28^

The detection of thyroid peroxidase antibodies (anti-TPO)^14,24^ and the presence of diffuse or nodular goiters on thyroid ultrasound point to AIT 1,^21,23^ nevertheless due to their high prevalence in the population, these findings also do not exclude AIT 2.^13,14,24^ Several recent studies indicate that the absence of hyperflow on Dopplerfluxometry is suggestive of AIT 2.^19,24,27,28^ However, these findings should not be used alone due to the possibility of mixed forms.^14^

### Maintain or discontinue amiodarone?

The need for amiodarone withdrawal is still controversial. In many cases, it is the only medication capable of controlling cardiac arrhythmia, and due to its prolonged half-life, the removal would not bring immediate benefits.^14^ In addition, it is important to note that some patients have a recurrence of thyroid disorders, even months after amiodarone interruption. Furthermore, the drug has T3 antagonist properties and inhibits the conversion of T4 to T3 in the heart, so its removal could aggravate the clinical manifestations.^20,21,24^

AIT 2 is generally self-limited, and amiodarone may be maintained in these patients.^14,29-32^ Observational studies with AIT 2 patients have shown that patients return to euthyroidism even when maintaining the amiodarone.^29,30,31^ However, studies show a variation of 8% to 73% of thyrotoxicosis recurrence in patients that continued using the medication.^29,31,33,34^ A 10-year follow-up study involving 50 patients who maintained amiodarone reported only three cases of thyrotoxicosis recurrence, much milder than in the first episode.^32^

The decision to withdraw amiodarone should be individualized and made jointly by the cardiologist and the endocrinologist, considering the risks and benefits of the drug withdrawal.^14,19,24^ Continuing the medication is widely accepted in critically ill patients with life-threatening arrhythmias who have a good cardiac response to the drug.^14,24,32^ If the cardiac conditions are stable and there is another safe alternative, amiodarone may be discontinued.^13,14^

### Treatment

Clinically stable patients with evidence that differentiates the treatment’s subtypes must be established according to the subtype in which the patient fits.^14,19,24^ In cases of mild thyrotoxicosis with impaired cardiac function, the American Thyroid Association recommends initiating combination therapy with antithyroid drugs and corticosteroids.^24^

If the patient presents with fast deterioration of cardiac function, emergency thyroidectomy should be performed regardless of AIT subtype.^14,24^ Figure 2 shows the algorithm for AIT management as proposed by the European Thyroid Association.^14^ Since the thyroid is loaded with iodine in AIT cases, radioactive iodine treatment is not feasible for at least six to nine months after the drugwithdrawal.^13,19,20^

**Figure 2:** Algorithm for the management of amiodarone-induced thyrotoxicosis (AIT). Modified from Bartalena L, et al.^14^

### Treatment: AIT 1

AIT 1 treatment is done with antithyroid drugs (ATD), but these are less effective due to the high iodine concentration, and it is necessary to use higher doses (40-60mg/day) methimazole or equivalent doses of propylthiouracil).^14,24^ If the patient remains stable, the ATD should be maintained until euthyroidism restoration,^14,19,20,35^ usually between three to six months.^24^

Potassium perchlorate may be associated in the first weeks to decrease thyroid uptake of iodine and make the thyroid more sensitive to the ATD.^1,14,19,35,36^ Due to its toxicity, it should not exceed 1g/day and should not be maintained for more than 4-6 weeks.^1,14^

The thyrotoxicosis may recur or may even not go into remission, and in these cases, the definitive treatment is recommended.^14,19,36^ If amiodarone is discontinued, definitive radioiodine treatment can be done after six to nine months. Thyroidectomy should be considered if amiodarone withdrawal is not possible.^14,19^ Overall, the definitive treatment of AIT 1 is similar to spontaneous hyperthyroidism.^14^

### Treatment: AIT 2

AIT 2 is usually self-limited, yet due to the increased mortality associated with thyrotoxicosis in cardiac patients, the treatment should be instituted to achieve euthyroidism more rapidly.^14,19,36^ The decision to treat mild or subclinical cases should be made considering the patient’s cardiac alterations.^14^

It has been suggested that, in well-defined AIT 2 cases, corticosteroid treatment is more effective than ATD treatment.^29,37^ The doses used are 30-40mg/day of prednisone or equivalent dose of another glucocorticoid for two to three months, with subsequent gradual withdrawal based on clinical response.^14,24^ In severe cases, as well as in AIT 1 and mixed/undefined AIT cases, radical thyroidectomy should be considered.^8,14,38^

### Treatment: TIA mixed or undefined

Mixed or undefined forms are not yet fully characterized. However, it is believed that these cases involve mixed pathogenic mechanisms of both subtypes, such as increased hormones production and destructive thyroiditis.^14,36^

The treatment of undefined forms should begin with ATD, and oral corticosteroids may be associated at the beginning of the treatment or after 4-6 weeks if the response is small.^14,19,35^ In more severe cases, combination therapy with ATD and corticosteroids should be promptly initiated.^24^

### Treatment: Thyroidectomy

Total thyroidectomy is the best option in patients whose clinical treatment is flawed or those with delayed therapeutic response associated with depressed ventricular function, which is currently the best alternative for immediate euthyroidism restoration.^14,39,40^ Despite the risks associated with thyroidectomy, it should be considered before the patient progresses with severe clinical worsening, as the delay in surgery indication is associated with increased mortality.^24,39-42^ Several studies evaluating AIT patients submitted to thyroidectomy reported low morbidity associated with the procedure, showing a 0% to 1.9% mortality rate.^40-44^

In a recent observational study, 207 AIT patients submitted to thyroidectomy (57 thyroidectomized, 156 clinical treatment) evidenced lower mortality in patients who underwent thyroidectomy than those who were only treated clinically, particularly in LVEF <40% patients. This same study demonstrated a significant improvement of LVEF after euthyroidism restoration, being more evident in LVEF <40% patients.^42^ Other three studies also reported significant improvement of cardiac function after thyroidectomy, being three patients removed from the cardiac transplant list after euthyroidism restoration.^40,41,43^

If total thyroidectomy is considered, individualized risk and benefit assessment should be made, and the decision should be multidisciplinary, involving cardiologists, endocrinologists, surgeons, and anesthesiologists. It is essential that a surgeon with high operative volume and experience with thyroidectomies is responsible for the procedure.^14^

Total thyroidectomy should be considered when:^14,24,39,43^

– Insufficient response to drug treatment with ATD and corticosteroids;
– Rapid deterioration of cardiac function;
– Advanced heart disease, right ventricular arrhythmogenic dysplasia, and malignant arrhythmias;
– Definitive treatment alternative to radioiodine (I^131^);

## Conclusion

Given the consequences caused by TIA, the importance of diagnosing and treating TIA subtypes together is highlighted. Furthermore, it emphasizes the importance of therapeutic decisions being taken jointly by cardiologists and endocrinologists and that in more severe cases, thyroidectomy should be considered before exaggerated clinical worsening occurs.

Clinical studies involving patients with AIT are still limited and insufficient, particularly multicenter randomized controlled trials. Since amiodarone is a widely used drug, and due to AIT consequences, the need for new clinical trials to improve the management of these patients is highlighted.
